# Supplementary material for: Axonemal regulation by curvature explains sperm flagellar waveform modulation
Source: PNAS Nexus. 2023 Mar 9;2(3):pgad072. doi: 10.1093/pnasnexus/pgad072 (PMC10063217; doi:10.1093/pnasnexus/pgad072)
Supplement: pgad072_Supplementary_Data [file pgad072_supplementary_data.pdf]

PNAS Nexus, 2021, pp. 1–4

doi: DOI HERE
Advance Access Publication Date: Day Month Year
Supplementary Material

SUPPLEMENTARY MATERIAL

Axonemal regulation by curvature explains sperm flagellar waveform modulation

Meurig T. Gallagher,[a,b,c,*] Jackson C. Kirkman-Brown[c] and David J. Smith[b,c]

[a]Centre for Systems Modelling and Quantitative Biomedicine, University of Birmingham, Birmingham B15 2TT, United Kingdom, [b]School of Mathematics, University of Birmingham, Birmingham B15 2TT, United Kingdom and [c]Centre for Human Reproductive Science, University of Birmingham and Birmingham Women's and Children's NHS Foundation Trust, Birmingham B15 2TT, United Kingdom
[*]To whom correspondence should be addressed: m.t.gallagher@bham.ac.uk

Abstract

Supplementary material for the manuscript: Axonemal regulation by curvature explains sperm falegllar waveform modulation.

Draft

© The Author 2021. Published by Oxford University Press. All rights reserved. For permissions, please e-mail: journals.permissions@oup.com

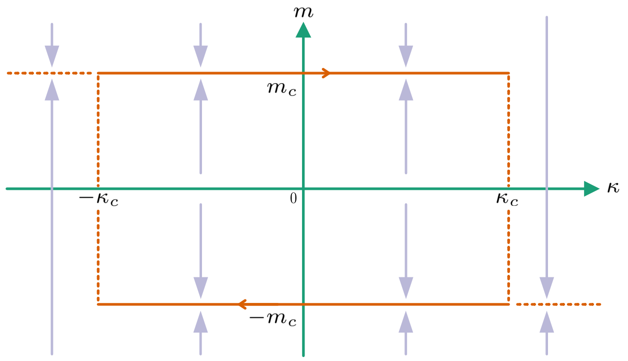

Fig. S1. Overview of the ARCH model showing a sketch of the active moment density m against curvature κ, for critical curvature threshold κ_c and preferred moment m_c. Arrows show the direction of the gradient of active moment density in the ARCH model.

S1 Derivation of the Axonemal Regulation of Curvature, Hysteretic model (ARCH)

To briefly recap the main manuscript, the underlying hypothesis for the ARCH model is the simple and elegant concept of *curvature control*. In simple terms, that the dynein activity within the axoneme will cause the sperm flagellum to bend, increasing curvature, until a threshold is reached whereupon the process will reverse. In the ARCH model, we adapt this hypothesis to one of *active moment control*, wherein we relate the rate of change of active moment density, m, to the curvature, κ (Fig. S1).

We derive the model by considering, in turn, the behavior of the top $m > 0$ and bottom $m < 0$ branches of the model respectively.

$m > 0$

A positive active moment density will cause a section of the axoneme to bend, thus increasing local curvature. We hypothesize that there is a preferred moment, m_c, owing to the activity of dynein and the arrangement of the axoneme. Therefore, while the curvature is less than some critical curvature threshold, $\kappa < \kappa_c$, the active moment density will approach the preferred moment as shown in Fig. S1. When the curvature is greater than the critical curvature threshold the active moment density will decrease to the negative preferred moment $-m_c$. These considerations allow us to write the following equation for the rate of change of active moment density when $m > 0$,

$$\tau_d \dot{m} = -\left(m - m_c\right)\mathcal{H}\left(\kappa_c - \kappa\right) - m_c\mathcal{H}\left(\kappa - \kappa_c\right), \quad m > 0, \tag{S1}$$

where τ_d^{-1} is a characteristic dynein switching rate, and $\mathcal{H}\left(\cdot\right)$ is the Heaviside function. We note that the first term on the left-hand side provides the attraction to the preferred moment, while the curvature threshold switching behavior comes from the second term.

$m < 0$

Similarly, but in reverse to the case $m > 0$, when $m < 0$ the active moment density will approach the negative preferred moment, $-m_c$, while the curvature κ is *greater* than the *negative* critical curvature threshold, $-\kappa_c$. When $\kappa < -\kappa_c$, the active moment density will *increase* to the preferred moment density m_c. We write,

$$\tau_d \dot{m} = -\left(m + m_c\right)\mathcal{H}\left(\kappa_c + \kappa\right) + m_c\mathcal{H}\left(-\kappa - \kappa_c\right), \quad m < 0. \tag{S2}$$

Combining for all m

Combining Eqns (S1) and (S2), we obtain

$$\tau_d \dot{m} = -\left(m - \mathrm{sgn}(m)\, m_c\right)\mathcal{H}\left(\kappa_c - \mathrm{sgn}(m)\kappa\right) - \mathrm{sgn}(m) m_c \mathcal{H}\left(-\left(\kappa_c - \mathrm{sgn}(m)\right)\kappa\right), \quad \forall m, \tag{S3}$$

where $\mathrm{sgn}(\cdot)$ is the signum function. Using the fact that $\mathcal{H}\left(x\right) = 1 - \mathcal{H}\left(-x\right)$, we obtain (after simplification) the dimensional ARCH model, i.e.

$$\tau_d \dot{m} = -\left(m - 2m_c \mathrm{sgn}(m)\right)\mathcal{H}\left(\kappa_c - \mathrm{sgn}\left(m\right)\kappa\right) - m_c \mathrm{sgn}\left(m\right), \quad \forall m. \tag{S4}$$

Generalizations of the ARCH model

In the present work we consider the preferred moment density and critical curvature thresholds at each switch to be of the same magnitude, differing only in sign. However, we have taken care to set up the model to allow (via the process outlined above), the choice of the thresholds to differ with only minor modifications to the model.

Draft

S2 Numerical discretisation

As highlighted in the main manuscript, the full system of equations for the ARCH model: Eqs. (1–2), and Eqs. (5–8) are discretised following the approach contained within Refs. [5, 6], with the non-local hydrodynamics accounted for using the parallelised NEAREST method of Smith and Gallagher [7, 2, 3, 4].

Head discretisation

The sperm head is modelled as an ellipsoidal surface, semi-axes lengths ($2\ \mu$m, $1.6\ \mu$m, $1\ \mu$m), inclined at an angle $\phi(t)$ to the horizontal, and discretised with $n_H = 24$ vector degrees of freedom (DOF) for the coarse force discretisation, denoted $\mathbf{Y}^{[n]}$ for $n = 1, \ldots, n_H$, and $n_Q = 384$ DOF for the fine quadrature discretisation, denoted $\hat{\mathbf{Y}}^{[n]}$ for $n = 1, \ldots, n_Q$. The force per unit area exerted by the head onto the fluid is denoted $\boldsymbol{\varphi}^{[n]} := \boldsymbol{\varphi}(\mathbf{Y}^{[n]}, t)$, $n = 1, \ldots, n_H$.

Flagellum discretisation

The flagellum is discretised into $n_T = 60$ straight-line segments of equal length $\Delta s = 1/n_T$. Segment end points are denoted $\mathbf{X}^{[n]}(t) := \mathbf{X}(s^{[n]}, t)$, at arclength $s^{[n]} := \Delta s$, for $n = 0, \ldots, n_T$ (where $\mathbf{X}^{[0]}$ is the point joining the head and flagellum). The angle joining $\mathbf{X}^{[n-1]}$ to $\mathbf{X}^{[n]}$ is denoted $\tilde{\theta}^{[n]}$ for $n = 1, \ldots, n_T$. Approximating the force density exerted by the flagellum on the fluid as piecewise constant, we write $\mathbf{f}(s,t) \approx \tilde{\mathbf{f}}^{[n]} = \mathbf{f}(\tilde{s}^{[n]}, t)$, with $\tilde{s}^{[n]} = (n - 1/2)\Delta s$ for $n = 1, \ldots, n_T$. The active moment is approximated as piecewise linear along each segment, with $m^{[n]} := m(s^{[n]}, t)$, $n = 0, \ldots, n_M - 1$ denoting the active moment at the actively bending segment joints. Here, $n_M = 57 < n_T$ has been chosen to represent the approximate 95% of the flagellum that actively bends.

Discretised system

The spatially discretised equations can then be written, for the force- and moment-free conditions as

$$\sum_{n=1}^{n_H} \varphi_j^{[n]} \sum_{m=1}^{n_Q} \delta_{ij} \nu^{[m,n]} + \sum_{n=1}^{n_T} \Delta s\, \tilde{f}_i^{[n]} = 0, \quad i = 1, 2; \tag{S5}$$

$$\delta_{i3} \sum_{n=1}^{n_H} \epsilon_{ijk} \left[Y_j^{[n]} - X_j^{[1]}\right] \varphi_k^{[n]} \sum_{m=1}^{n_Q} \delta_{ij} \nu\,[m, n] + \sum_{n=1}^{n_T} \Delta s \epsilon_{ijk} \left[\tilde{X}_j^{[n]} - X_j^{[1]}\right] \tilde{f}_k^{[n]} = 0; \tag{S6}$$

for the elastic behaviour of the flagellum as

$$E\left(s^{[n]}\right) \left[\frac{\tilde{\theta}^{[n+1]} - \tilde{\theta}^{[n]}}{\Delta s}\right] - \mathcal{M}\Delta s \left[\frac{1}{2} m^{[n]} + \sum_{i=n+1}^{n_M - 1} m^{[i]}\right] = -\mathcal{S}^4 \mathbf{e}_3 \cdot \sum_{i=n}^{n_T - 1} \Delta s \left[\tilde{\mathbf{X}}^{[i+1]} - \mathbf{X}^{[n]}\right] \times \tilde{\mathbf{f}}^{[i+1]}, \ n = 1, \ldots, n_M; \tag{S7}$$

$$E\left(s^{[n]}\right) \left[\frac{\tilde{\theta}^{[n+1]} - \tilde{\theta}^{[n]}}{\Delta s}\right] = -\mathcal{S}^4 \mathbf{e}_3 \cdot \sum_{i=n}^{n_T - 1} \Delta s \left[\tilde{\mathbf{X}}^{[i+1]} - \mathbf{X}^{[n]}\right] \times \tilde{\mathbf{f}}^{[i+1]}, \ n = n_M, \ldots, n_T - 1; \tag{S8}$$

$$E\left(s^{[0]}\right) \left[\frac{\tilde{\theta}^{[1]} - \phi}{\Delta s}\right] - \mathcal{M}\Delta s \left[\frac{1}{2} m^{[0]} + \sum_{i=1}^{n_M - 1} m^{[i]}\right] = -\mathcal{S}^4 \mathbf{e}_3 \cdot \sum_{i=0}^{n_T - 1} \Delta s \left[\tilde{\mathbf{X}}^{[i+1]} - \mathbf{X}^{[0]}\right] \times \tilde{\mathbf{f}}^{[i+1]}; \tag{S9}$$

for the hydrodynamic equations evaluated for points on the flagellum, with $n = 1, \ldots, n_T$, as

$$\begin{aligned} &\dot{X}_i^{[0]} + \mathbf{e}_i \cdot \left[\frac{\Delta s}{2} \dot{\tilde{\theta}}^{[n]} \left[-\sin \tilde{\theta}^{[n]}, \cos \tilde{\theta}^{[n]}, 0\right]^T + \sum_{k=1}^{n-1} \Delta s\, \dot{\tilde{\theta}}^{[k]} \left[-\sin \tilde{\theta}^{[k]}, \cos \tilde{\theta}^{[k]}, 0\right]^T\right] \\ &\quad = \sum_{k=1}^{n_H} \varphi_j^{[k]} \sum_{l=1}^{n_Q} S_{ij}^{\varepsilon} \left(\tilde{\mathbf{X}}^{[n]}, \hat{\mathbf{Y}}^{[l]}\right) \nu^{[k,l]} + \sum_{k=1}^{n_T} I_{ij}^{[k]} \left(\tilde{\mathbf{X}}^{[n]}, t; \Delta s, \varepsilon\right) \tilde{f}_j^{[k]}, \ i = 1, 2, 3; \end{aligned} \tag{S10}$$

where the integral

$$I_{ij}^{[k]}\left(\chi, t; \Delta s, \varepsilon\right) = \int_{(k-1)\Delta s}^{k\Delta s} S_{ij}^{\varepsilon} \left(\chi, \tilde{\mathbf{X}}^{[k]} + \left(s - \tilde{s}^{[k]}\right) \left[\cos \tilde{\theta}^{[k]}, \sin \tilde{\theta}^{[k]}, 0\right]^T\right) \mathrm{d}s, \tag{S11}$$

is calculated analytically following Refs. [8, 1, 5]; the hydrodynamic equations evaluated for points on the head, with $n = 1, \ldots, n_H$, are written as

$$\mathbf{e}_i \cdot \left[\dot{\mathbf{X}}^{[0]} + \dot{\phi}\, \mathbf{e}_3 \times \left[\mathbf{Y}^{[n]} - \mathbf{X}^{[0]}\right]\right] = \sum_{k=1}^{n_H} \varphi_j^{[k]} \sum_{l=1}^{n_Q} S_{ij}^{\varepsilon} \left(\mathbf{Y}^{[n]}, \hat{\mathbf{Y}}^{[l]}\right) \nu^{[k,l]} + \sum_{k=1}^{n_T} I_{ij}^{[k]} \left(\mathbf{Y}^{[n]}, t; \Delta s, \varepsilon\right) \tilde{f}_j^{[k]}, \ i = 1, 2, 3; \tag{S12}$$

for the rate equation governing the active moment, for $n = 1, \ldots, n_M$, as

$$\dot{m}^{[n]} = -\left(m^{[n]} - 2\,\mathrm{sgn}\left(m^{[n]}\right)\right) \mathcal{H}\left(\kappa_c - \mathrm{sgn}\left(m^{[n]}\right) \frac{\tilde{\theta}^{[n+1]} - \tilde{\theta}^{[n]}}{\Delta s}\right) - \mathrm{sgn}\left(m^{[n]}\right). \tag{S13}$$

Draft

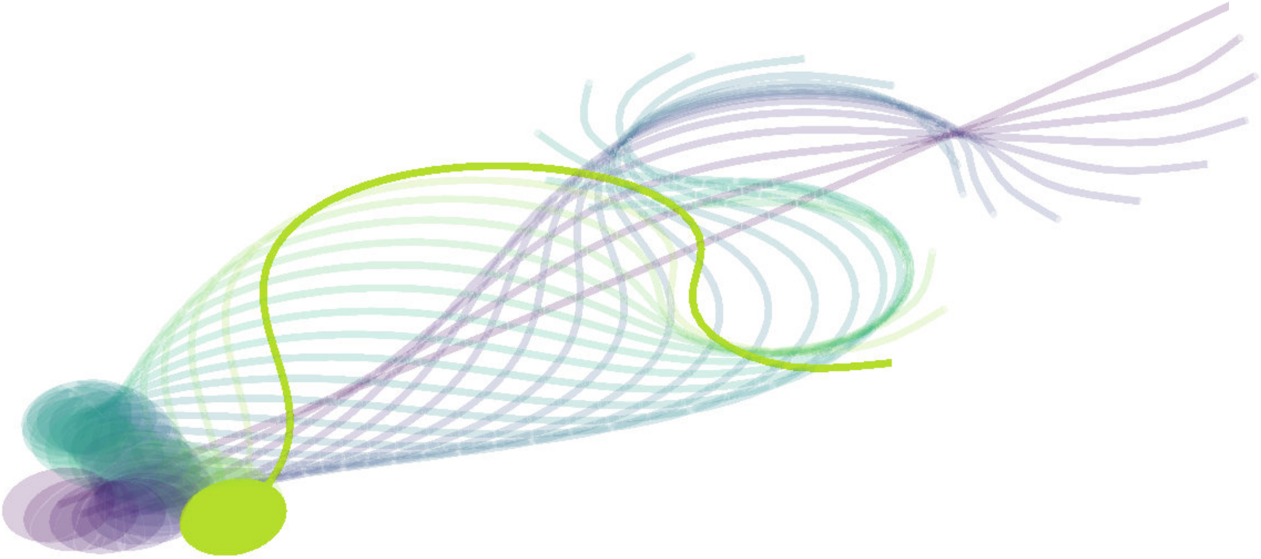

Fig. S2. Evolution of the flagellar beat from the initial low-amplitude parabola.

The spatially discrete system can then be written in the form of a non-linear autonomous initial value problem,

$$\dot{\mathbf{z}} = \mathcal{F}(\mathbf{z}), \qquad \boldsymbol{z}(0) = \boldsymbol{z}_0, \tag{S14}$$

where $\dot{\mathbf{z}} = \partial \boldsymbol{z}/\partial t$, with $\mathbf{z}(t) := \left[\mathbf{X}^{[1]}(t), \phi(t), \tilde{\theta}^{[1]}, \ldots, \tilde{\theta}^{[n_T]}, \tilde{m}^{[1]}(t), \ldots, \tilde{m}^{[n_M]}(t)\right]^T$. The system (S14) can be augmented at each time point to include the unknown force densities as

$$A \begin{bmatrix} \dot{\mathbf{z}} \\ \mathbf{f} \\ \boldsymbol{\varphi} \end{bmatrix} = \mathbf{b}, \tag{S15}$$

with

$$A = \left[\begin{array}{c|c|c} 0 & A_E & 0 \\ \hline A_K & A_H & 0 \\ \hline 0 & 0 & A_M \end{array}\right]. \tag{S16}$$

The matrix blocks of A (A_E, A_K, A_H, A_M) encode the elastodynamic, force- and moment-free equations (Eqs. S5–S9); the kinematic and hydrodynamic equations (Eqs. S10–S12); and active moment equations ((S13)) respectively.

References

1. M.T. Gallagher, T.D. Montenegro-Johnson, and D.J. Smith. Simulations of particle tracking in the oligociliated mouse node and implications for left–right symmetry-breaking mechanics. *Phil. Trans. Roy. Soc. B*, 375(1792):20190161, 2020.
2. M.T. Gallagher and D.J. Smith. Meshfree and efficient modeling of swimming cells. *Phys. Rev. Fluids*, 3(5):053101, 2018.
3. M.T. Gallagher and D.J. Smith. Passively parallel regularized stokeslets. *Phil. Trans. R. Soc. A*, 378(2179):20190528, 2020.
4. M.T. Gallagher and D.J. Smith. The art of coarse stokes: Richardson extrapolation improves the accuracy and efficiency of the method of regularized stokeslets. *Roy. Soc. Open Sci.*, 8(5):210108, 2021.
5. A.L. Hall-McNair, T.D. Montenegro-Johnson, H. Gadêlha, D.J. Smith, and M.T. Gallagher. Efficient implementation of elastohydrodynamics via integral operators. *Phys. Rev. Fluids*, 4(11):113101, 2019.
6. C.V. Neal, A.L. Hall-McNair, J. Kirkman-Brown, D.J. Smith, and M.T. Gallagher. Doing more with less: The flagellar end piece enhances the propulsive effectiveness of human spermatozoa. *Phys. Rev. Fluids*, 5(7):073101, 2020.
7. D.J. Smith. A nearest-neighbour discretisation of the regularized stokeslet boundary integral equation. *J. Comput. Phys.*, 358:88–102, 2018.
8. D.J. Smith, E.A. Gaffney, H. Gadêlha, N. Kapur, and J.C. Kirkman-Brown. Bend propagation in the flagella of migrating human sperm, and its modulation by viscosity. *Cell Motil. Cytoskel.*, 66(4):220–236, 2009.

Draft

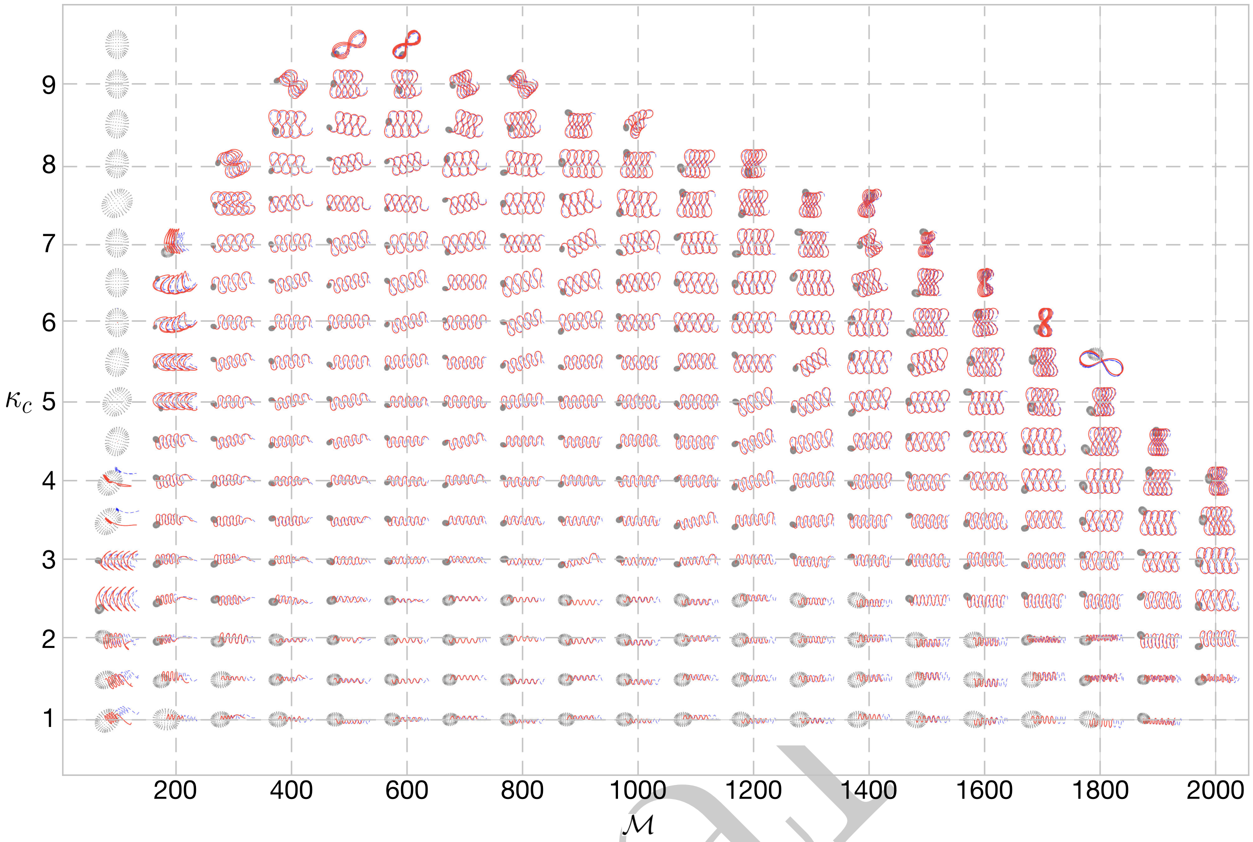

Fig. S3. Sperm tracks from simulations plotted across the regulatory parameter subspace $(\mathcal{M}, \kappa_c)$. The active moment $(\mathcal{M})$ and critical curvature (κ_c) parameters are varied with $(\mathcal{S}, \rho) = (18, 36.4)$. Parameter choices with missing entries (e.g. $(\mathcal{M}, \kappa_c) = (100, 5)$) are unable to generate a beat. Red solid lines show the path traced about by the centroid of the head, while the blue dashed curves show the path of the head/flagellum join. Each simulation is shown for a period of 5 complete beat cycles. Sperm shown with heads only have a stationary flagellum.

Draft

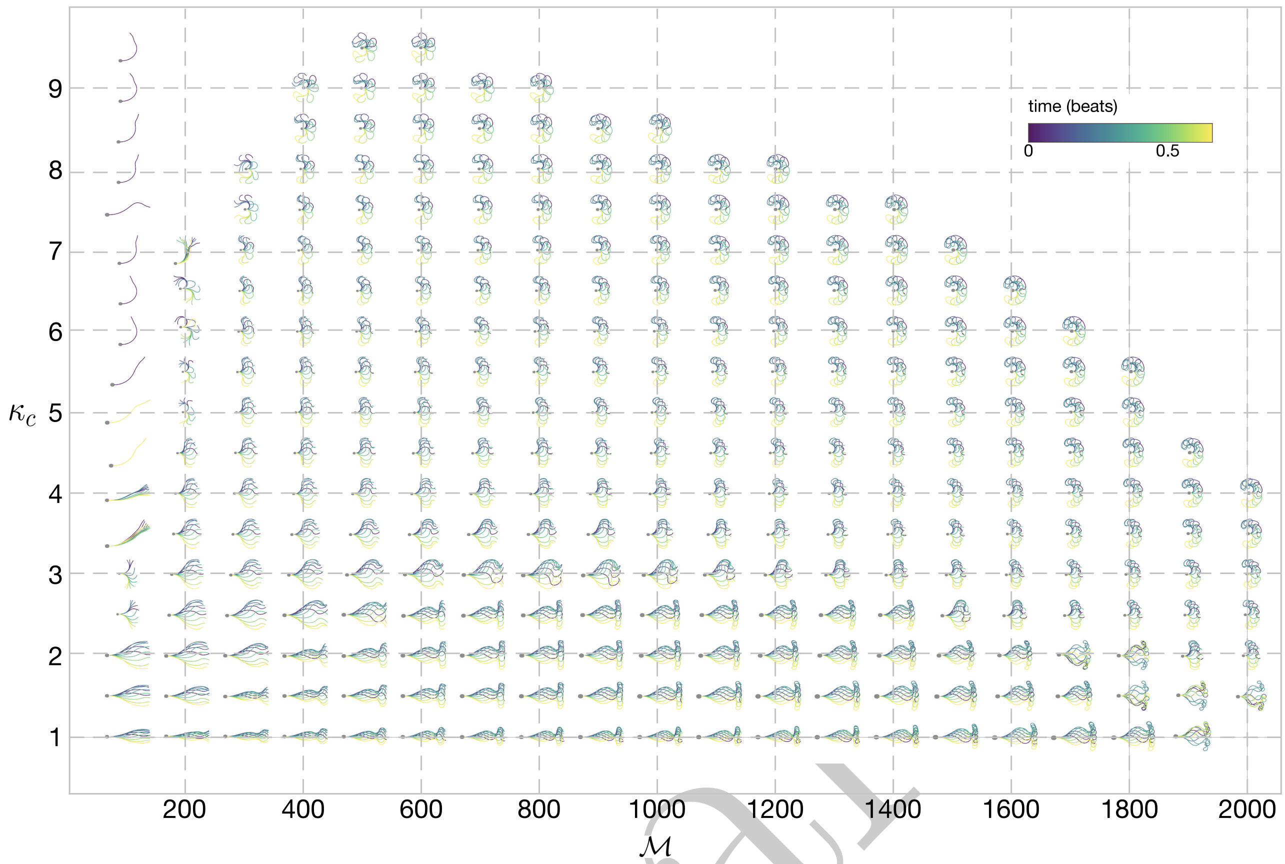

Fig. S4. Sperm waveforms from simulations plotted across the regulatory parameter subspace $(\mathcal{M}, \kappa_c)$. The active moment $(\mathcal{M})$ and critical curvature (κ_c) parameters are varied with $(\mathcal{S}, \rho) = (18, 36.4)$. Parameter choices with missing entries (e.g. $(\mathcal{M}, \kappa_c) = (100, 5)$) are unable to generate a beat.

Draft

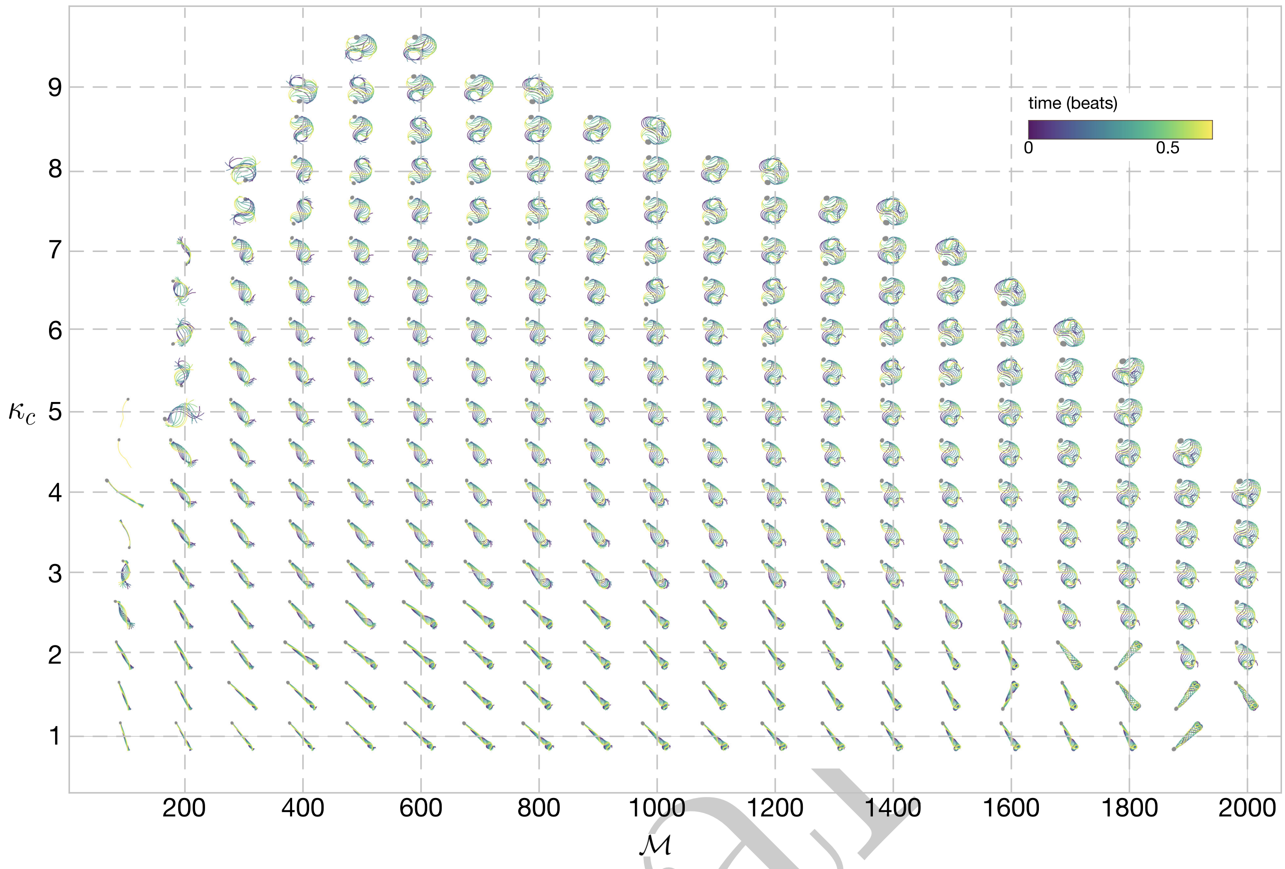

Fig. S5. Sperm flagellar time-lapse from simulations plotted across the regulatory parameter subspace $(\mathcal{M}, \kappa_c)$. The active moment $(\mathcal{M})$ and critical curvature (κ_c) parameters are varied with $(\mathcal{S}, \rho) = (18, 36.4)$. Parameter choices with missing entries (e.g. $(\mathcal{M}, \kappa_c) = (100, 5)$) are unable to generate a beat.

Draft

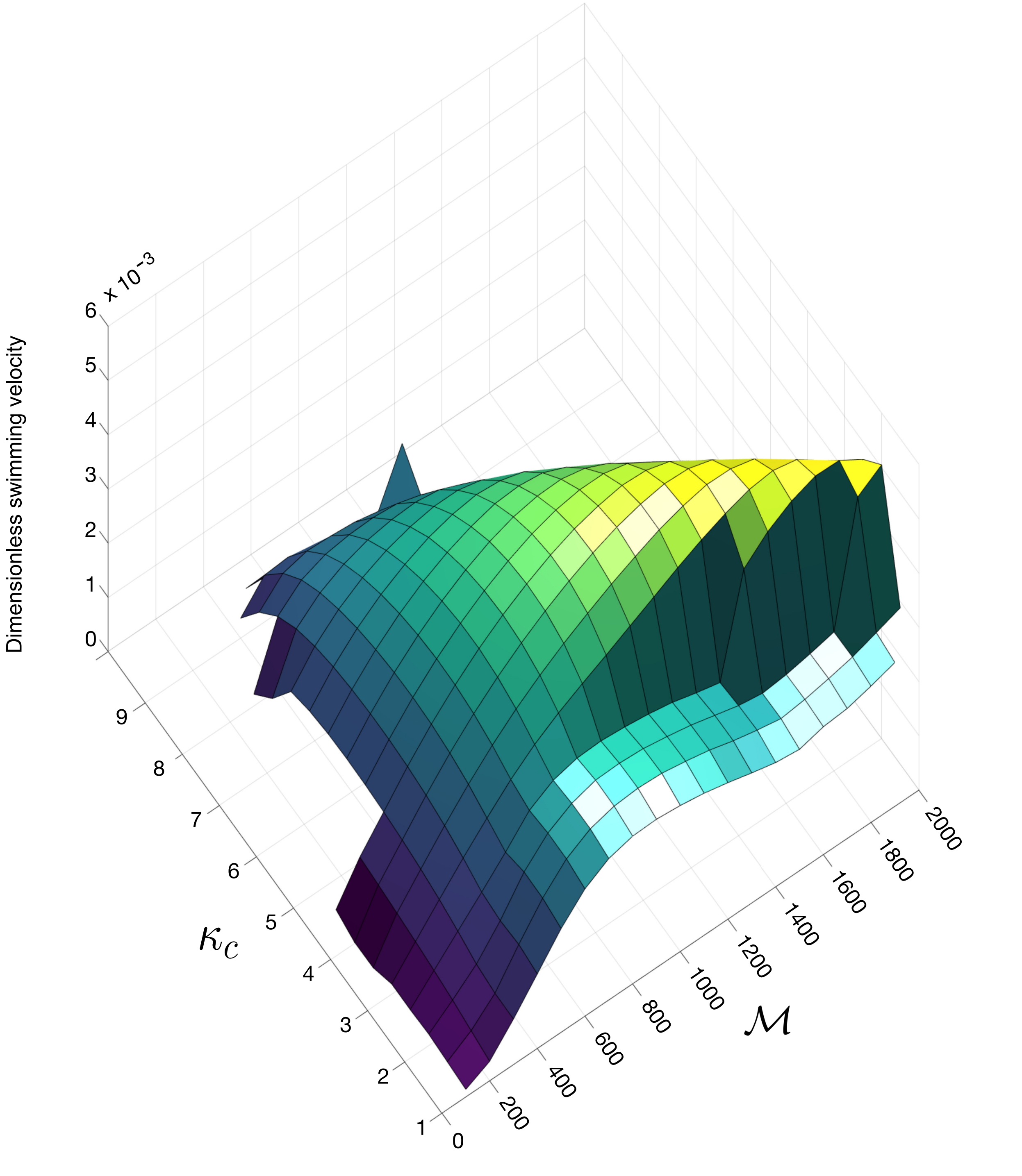

Fig. S6. Dimensionless swimming velocity of sperm simulations plotted across the regulatory parameter subspace $(\mathcal{M}, \kappa_c)$. The active moment $(\mathcal{M})$ and critical curvature (κ_c) parameters are varied with $(\mathcal{S}, \rho) = (18, 36.4)$. Parameter choices with missing entries (e.g. $(\mathcal{M}, \kappa_c) = (100, 5)$) are unable to generate a beat.

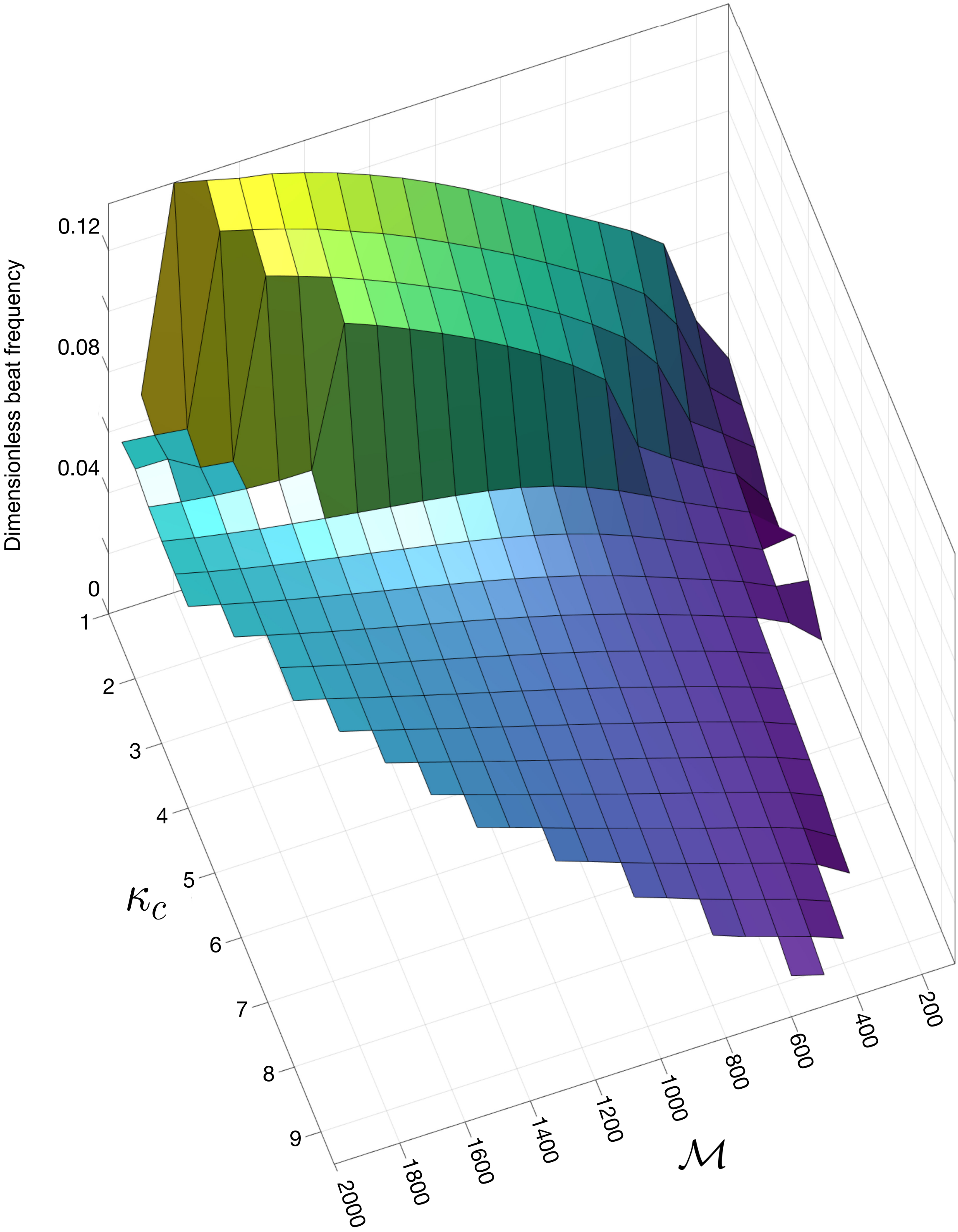

Fig. S7. Dimensionless flagellar beat frequency of sperm simulations plotted across the regulatory parameter subspace $(\mathcal{M}, \kappa_c)$. The active moment $(\mathcal{M})$ and critical curvature (κ_c) parameters are varied with $(\mathcal{S}, \rho) = (18, 36.4)$. Parameter choices with missing entries (e.g. $(\mathcal{M}, \kappa_c) = (100, 5)$) are unable to generate a beat.

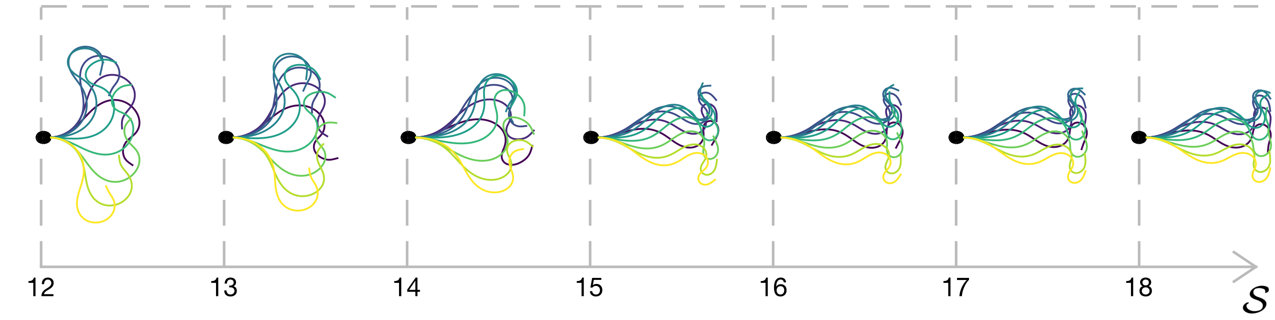

Fig. S8. Sperm waveforms from simulations plotted for changes in the viscous/elastic ratio $\mathcal{S}$, varied across $\mathcal{S} \in [12, 18]$ for the choice $(\mathcal{M}, \kappa_c, \rho) = (800, 2, 36.4)$.

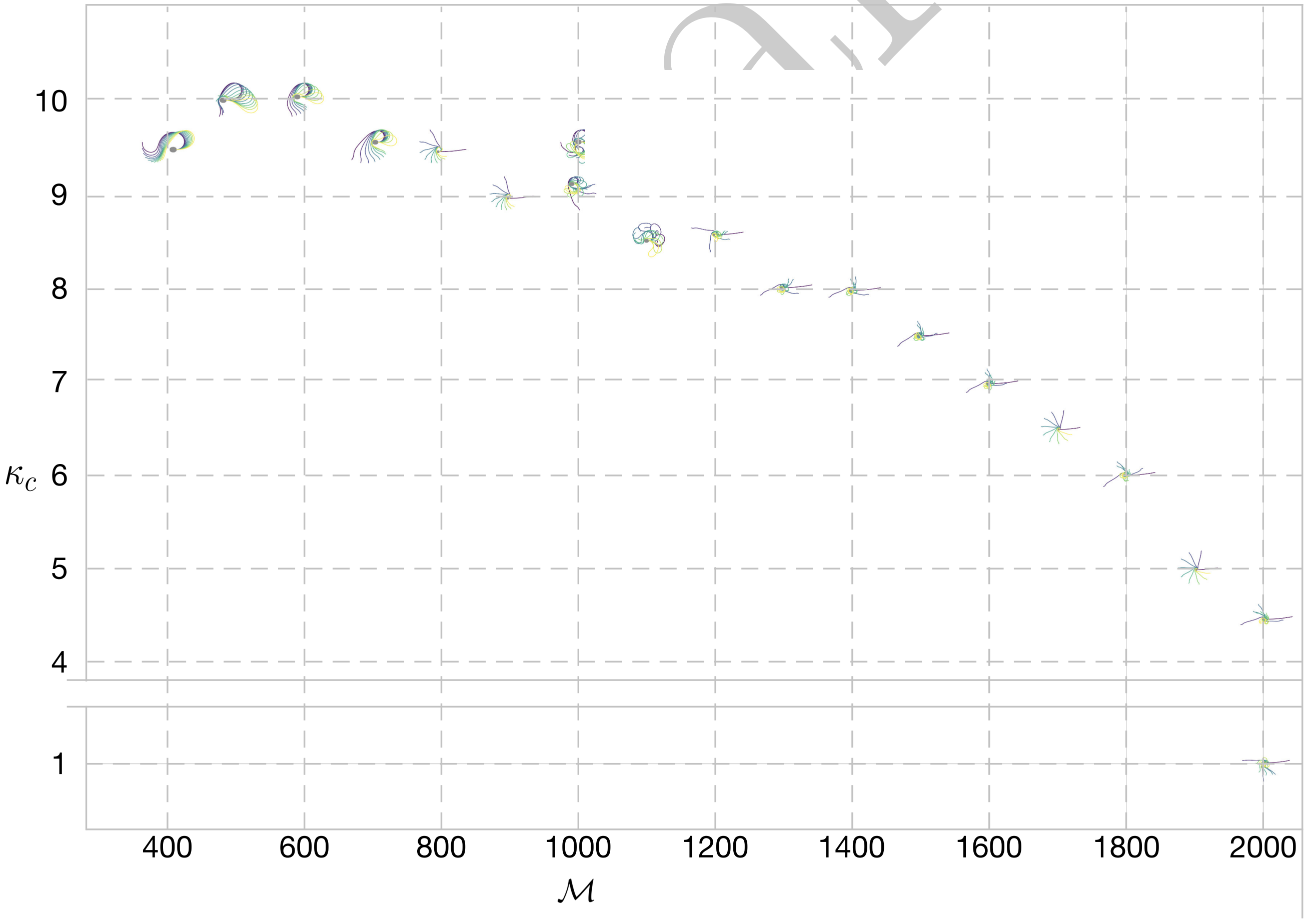

Fig. S9. Sperm waveforms from simulations that failed to solve due to self-intersection, plotted across the regulatory parameter subspace $(\mathcal{M}, \kappa_c)$. The active moment $(\mathcal{M})$ and critical curvature (κ_c) parameters are varied with $(\mathcal{S}, \rho) = (18, 36.4)$.
